# Electroencephalographic slow wave dynamics and loss of behavioural responsiveness induced by ketamine in human volunteers

**DOI:** 10.1016/j.bja.2019.07.021

**Published:** 2019-09-03

**Authors:** Jamie Sleigh, Rebecca M. Pullon, Phillip E. Vlisides, Catherine E. Warnaby

**Affiliations:** 1 Department of Anaesthesia, Waikato Clinical Campus, University of Auckland, Hamilton, New Zealand; 2 Department of Anesthesiology, University of Michigan Medical School, Ann Arbor, MI, USA; 3 Wellcome Centre for Integrative Neuroimaging, Nuffield Department of Clinical Neurosciences, University of Oxford, John Radcliffe Hospital, Headington, Oxford, UK

**Keywords:** consciousness, electroencephalography, general anaesthesia, ketamine, pharmacodynamics, pharmacokinetics, slow wave activity

## Abstract

**Background:**

Previous work on the electroencephalographic (EEG) effects of anaesthetic doses of ketamine has identified a characteristic signature of increased high frequency (beta–gamma) and theta waves alternating with episodic slow waves. It is unclear which EEG parameter is optimal for pharmacokinetic–pharmacodynamic modelling of the hypnotic actions of ketamine, or which EEG parameter is most closely linked to loss of behavioural responsiveness.

**Methods:**

We re-analysed previously published 128-channel scalp EEG data from 15 subjects who had received a 1.5 mg kg^−1^ bolus i.v. dose of ketamine. We applied standard sigmoid pharmacokinetic–pharmacodynamic models to the drug-induced changes in slow wave activity, theta, and beta–gamma EEG power; and examined the morphology of the slow waves in the time domain for Fz, F3, T3, P3, and Pz average-referenced channels.

**Results:**

Hypnotic doses of ketamine i.v. induced medio-frontal EEG slow waves, and loss of behavioural response when the estimated brain concentration was 1.64 (0.17) μg ml^−1^. Recovery of responsiveness occurred at 1.06 (0.21) μg.ml^−1^ after slow wave activity had markedly diminished. Pharmacokinetic–pharmacodynamic modelling fitted best to the slow wave activity and theta power (almost half the beta–gamma channels could not be modelled). Slow wave effect-site equilibration half-time (23 [4] s), and offset, was faster than for theta (47 [22] s).

**Conclusions:**

Changes in EEG slow wave activity after a hypnotic dose of ketamine could be fitted by a standard sigmoid dose-response model. Their onset, but not their offset, was consistently associated with loss of behavioural response in our small study group.

**Editor's key points**

• There is considerable interest in electroencephalographic signatures for identifying the cortical effects of various anaesthetics.
• The EEG parameters correlating with pharmacokinetic–pharmacodynamic modelling of the hypnotic actions of ketamine were studied in human volunteers.
• Changes in slow wave activity after a hypnotic dose of ketamine were well fitted by a standard sigmoid model
• Onset, but not offset, of slow wave activity was consistently associated with loss of behavioural responsiveness.

The electroencephalographic (EEG) effects of ketamine have been studied for more than 50 yr.^1^ In an early paper, Schwartz and colleagues^1^ reported that hypnotic doses of ketamine produced strong increases in high frequency (beta–gamma, 20–45 Hz) and theta (4–8 Hz) waves, punctuated by episodic slow (delta, 0.25–1.5 Hz) waves (see Fig 1, Fig 2 in their paper, and subsequent work by Schüttler and colleagues^2^). Using multi-spectral techniques on EEG obtained from frontal electrodes, Akeju and co-workers^3^ have recently rediscovered, and more elegantly quantified, the ketamine-induced EEG pattern, in which bursts of high frequency activity alternate with slow waves. They termed this a ‘gamma burst’ pattern. However, they had only looked at frontal electrodes, and had also given most of the patients adjunctive midazolam and fentanyl. They suggested further work with high-density EEG collection needed to be done. Using such a system, a previous paper by Vlisides and colleagues^4^ compared sub-hypnotic infusions of ketamine with hypnotic doses, and found strikingly increased EEG theta power (with regional phase locking), decreased alpha power, and loss of anterior-to-posterior alpha connectivity. They also noted an increase in delta power.

**Fig 1:**
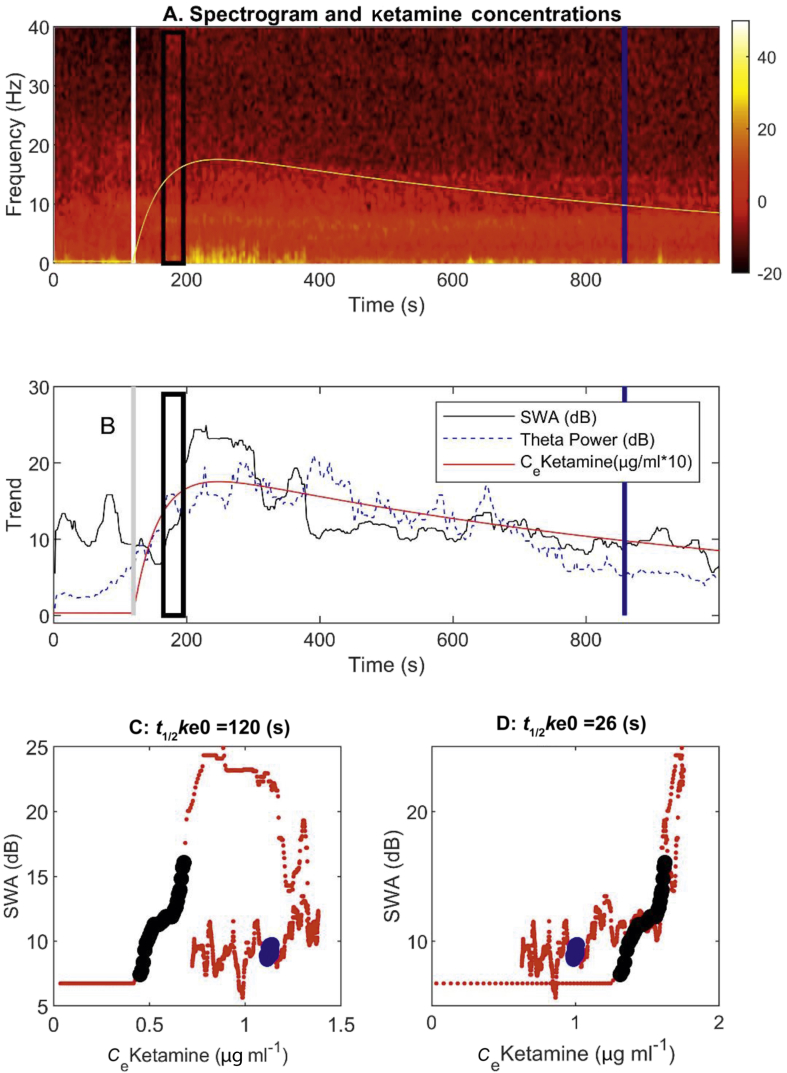
Changes in spectral power in response to intravenous injection of 1.5 mg kg^−1^ ketamine (administered at the white/grey vertical lines) for one individual. Times of loss of behavioural response (LOBR; black rectangle) and recovery of behavioural response (ROBR; blue line). We use a rectangle for LOBR because the subject could have lost responsiveness at any time in the 30 s window between the verbal commands from the audio loop. (a) Spectrogram of the EEG (yellow is high power, red medium power, and black low power). The green line shows the time course of the calculated effect-site concentration of ketamine (μg ml^−1^×10). (b) Time course of the slow wave activity (SWA) and theta power, and (c, d) examples of the hysteresis loops of effect-site concentrations *vs* SWA for two different *t*_1/2_*k*_e0_ values, where *t*_1/2_*k*_e0_ is the half-time of equilibration between blood and effect-site. LOBR and ROBR could have occurred over a 30 s period, hence the black and blue lines. Data are from channel 52, which corresponds to P3 in the 10–20 system.

**Fig 2:**
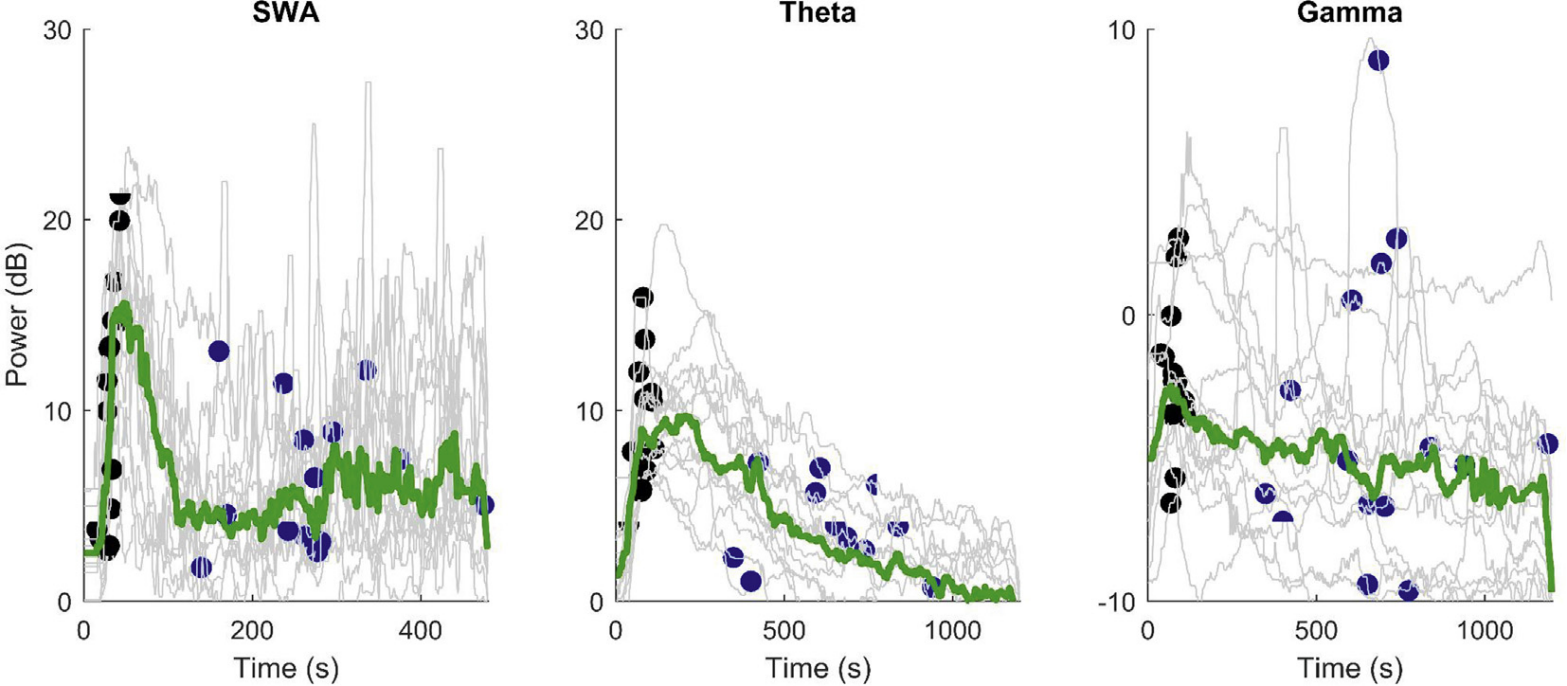
Time course of slow wave activity, theta, and beta–gamma power after induction (time=0 s) for channel 46 (T3) for all subjects. Individual trajectories are in grey, and the thick green line is the median at each time point. The black dots are the point of detection of loss of behavioural response to command, and the blue dots are the point of recovery of behavioural response to command. (For graphical clarity, dots have been used—the actual points of change in behavioural response could have occurred up to 30 s before the dots.)

Slow wave power is associated with loss of perception in subjects given gamma aminobutyric acid (GABA)-ergic drugs such as propofol or volatile anaesthetics.^5^, ^6^ Ketamine has different molecular targets; but if the EEG slow waves produced by ketamine are associated with loss of responsiveness, it suggests that slow waves might be causally mediating change in consciousness. A few pharmacokinetic–pharmacodynamic (PKPD) studies of ketamine have been reported, mainly looking at the perceived analgesic effect of ketamine^7^ or clinical sedation^8^ as the primary outcome. It is unclear which EEG parameter is optimal for the hypnotic PKPD modelling of ketamine, or which EEG parameter is most closely linked to loss of behavioural responsiveness (LOBR). The median frequency of the EEG has been related to serum ketamine concentrations using PKPD models.^2^ This is a composite measure, reflecting the balance between the divergent ketamine effects on the EEG, namely ketamine-induced increased slow wave power and ketamine-induced increase in higher frequency power. We performed a formal PKPD analysis of the high density EEG and ketamine data exploring dose-related changes in theta, beta–gamma, and slow wave activity (SWA). We hypothesised that the changes in SWA with a hypnotic dose of ketamine could be fitted by a standard sigmoid PKPD model, and their onset could be consistently associated with LOBR.

## Methods

### Data collection

The details of the data collection have been published.^4^, ^9^ In brief, after written informed consent and approval by the University of Michigan Medical School Institutional Review Board, Ann Arbor, MI, USA (HUM00061087), 15 volunteers (7 male/8 female, American Society of Anesthesiologists [ASA] physical status 1, 20–40 yr of age, BMI <30 kg m^−2^) were given a sub-anaesthetic i.v. infusion of ketamine 0.5 mg kg^−1^ administered over 40 min, followed by a 30 min pause for rest and psychometric testing.^9^ Then a hypnotic induction i.v. bolus dose of ketamine 1.5 mg kg^−1^ was administered. The times of loss and recovery of behavioural responsiveness (LOBR/ROBR) were estimated by an audioloop command to squeeze the right or left hand every 30 s. The EEG was obtained using 128-channel system (HydroCel nets, Net Amps 400 amplifiers, and Net Station 4.5 software; Electrical Geodesics, Inc., Eugene, OR, USA) and digitised at 500 Hz using a vertex reference.

### Signal processing and analysis

Basic spectral and connectivity patterns have been published for the first 10 participants in this study across both sedative and anaesthetic periods.^4^ Here, we report a separate PKPD analysis on the second (bolus) part of the study, where all participants (*n*=15) lost responsiveness. This occurred 30 min after cessation of the low-dose sub-hypnotic infusion. All processing was done using the Chronux (http://chronux.org/) and EEGLAB^10^ toolboxes, and purpose-written Matlab (MathWorks, Natick, MA, USA) scripts. The output of the EEG system produces a virtual DC signal. Channels were therefore re-referenced to an average reference, down-sampled to 125 Hz, and bandpass-filtered (0.1–50 Hz) using a fifth-order Butterworth filter and the ‘filtfilt.m’ phase preserving filter function. The spectral power (in dB) was calculated using the Chronux ‘mtspecgramc.m’ function on a moving 4 s segment of data (3 s overlap), time–bandwidth product of two and three tapers. SWA was calculated as mean power from 0.25 to 1.5 Hz, and this was smoothed using a median filter. Maximum theta (4–8 Hz) and beta–gamma (20–45 Hz) power were similarly calculated.

### Pharmacokinetic-pharmacodynamic modelling

Effect-site concentrations of ketamine were estimated using parameters derived from published work.^11^ Because the previous low-dose infusion will have slightly loaded the peripheral compartment, the time course of concentrations were calculated on the basis of whole ketamine administration (i.e. both the sub-hypnotic infusion and hypnotic bolus). The concentration of ketamine at the start of the hypnotic bolus was at a level that has no hypnotic effects (39 ng ml^−1^). To see if the results were robust to the choice of pharmacokinetic model, they were also analysed using the model of Clements and Nimmo.^12^ The results were not significantly different (*P*=0.47). In a separate unpublished pilot study, blood samples for plasma ketamine were obtained at the end of the slow infusion in seven similar subjects. The mean ketamine concentration was 183 ng ml^−1^, which was within the 95% confidence limits of the mean calculated using our PKPD model (124–195 ng ml^−1^). This confirmed that our PKPD model was well calibrated to the population mean. However, as in all PKPD modelling, individual variability in plasma concentrations was large. In the pilot study, the actual ketamine concentrations ranged from 62 to 440 ng ml^−1^.

To see if different regions of the brain showed different sensitivity to ketamine, we compared PKPD models of SWA from five 10–20 system channels (Fz, F3, T3, P3, Pz) chosen to represent medial, lateral, frontal, and parietal cortices. We also compared the PKPD modelling of theta and beta–gamma band powers with that of the SWA. For the modelling, we used 480 s of EEG data from the time of ketamine injection to fit the PKPD model. This time frame was chosen to concentrate on the period of unresponsiveness and minimise the various movement artifacts present around the time of ROBR. For the same reason, we set the baseline SWA, theta, and beta–gamma as the minimum for the period between ketamine injection and LOBR.

Modelling was done in three stages. Firstly, for each subject, channel, and frequency band, we ran an individual PKPD model (‘nlinfit.m’) using 50 different *k*_e0_ values equidistantly spaced on a logarithmic scale. This resulted in values for the half-time for equilibration between blood and effect-site (*t*_1/2_*k*_e0_) ranging from 8 to 139 s. Thus, we obtained the *k*_e0_ value for each subject and channel that gave the best model fit to the SWA, theta, and beta–gamma power as measured by coefficient of variation (*R*^2^). This effectively identified the *k*_e0_ required to collapse the hysteresis loop for each frequency band of interest (see Fig. 1c and d for SWA).

The drug effect on EEG power was fitted for each frequency band using a standard sigmoid function:

(1) $$\text{EEG}\;\text{power}=\beta_{1}+\frac{\beta_{2}}{1+\text{exp}\left(-\frac{C_{\text{e}}\text{Ketamine}-\beta_{3}}{\beta_{4}}\right)},$$

where *β*_1_ determines the baseline SWA, *β*_2_ the plateau SWA, *β*_3_ and *β*_4_ control the slope and position of the sigmoid, and *C*_e_Ketamine is the effect-site concentration of ketamine. The time courses of both the raw data and the fitted model were then overlaid and checked to confirm that the model produced a single peaked function that plausibly tracked the raw data and ketamine concentrations before entry into the mixed-effects model. This was because of convergence problems in the mixed-effects model if a significant proportion of the fits followed unphysiological trajectories.

The second stage of analysis was to estimate the sigmoid parameters using a non-linear mixed-effects regression model (‘nlmefitsa.m’) that used a stochastic expectation maximisation algorithm.^13^ Population parameters describing drug concentration *vs* EEG power (i.e. SWA/theta/beta–gamma) were included as fixed effects. Between-subject variation around the population means was included as a random effect. We also used a constant error model.

Finally, we statistically compared parameters and goodness-of-fit for different channels, subjects, and frequency bands using analysis of variance (anova).

### Slow wave morphology

The alternating delta–gamma burst pattern reported by Akeju and colleagues^3^ suggested an episodic phenomenon that might not necessarily be captured well using these frequency domain methods. We therefore also examined the morphology of individual slow waves in the time domain. To minimise filter-induced distortion of the waveform, we removed the baseline drift by subtracting a 4 s median filtered waveform. We then identified the time, width, and amplitude of any slow waves that had an amplitude of >4 inter-quartile ranges (IQRs) from baseline, a duration of >0.04 s and were not within 1.6 s of the previous wave.

### Statistical analysis

For normally distributed data (probability distribution tested using the Kolmogorov–Smirnoff test), we report observational data as mean (standard deviation, sd). To examine the influence of subject, channel, and frequency band on the model parameters and *R*^2^ values, we used single anova, with subject as the between-subject group, and channels and frequency bands (modelled as within-subject fixed effects). We used the Bonferroni test for *post hoc* group comparisons. Otherwise, we report median (IQR) and use the Wilcoxon rank sum test (or sign rank test for paired data) for comparisons.

## Results

In response to a hypnotic dose of ketamine, the EEG patterns for all subjects were similar to those described.^1^, ^2^, ^3^ A typical spectrogram is shown in Figure 1a, and demonstrates increased broadband gamma and beta power, and the appearance of narrowband theta oscillations after ketamine injection (white vertical line). A strong period of SWA can also be seen from around the point of loss of behavioural response (black rectangle) to about 400 s (Fig. 1b).

The hysteresis curves for SWA for two example *k*_e0_s (Fig. 1c and d) show that the collapse of the curve occurs with a shorter *t*_1/2_*k*_e0_ of 26 s. Overall, the best fit was achieved with mean *t*_1/2_*k*_e0_ for SWA of 23 (4) s (range 13–39 s). The *t*_1/2_*k*_e0_ for beta–gamma power was variable (mean 98 [72] s, range 9–138 s), and there were 33 out of 75 channels for which no good fit could be obtained. Subject (*P*<0.001), frequency band (*P*<0.001), and channel (*P*=0.002) all significantly influenced the *t*_1/2_*k*_e0_. In the *post hoc* analysis, *t*_1/2_*k*_e0_ for theta power was significantly longer (47 [22] s, range 13–138 s) than those of SWA and beta–gamma, which reflects the slower onset and offset of theta power. As regards channel effects, the only significant effect was that the *t*_1/2_*k*_e0_ for T3 was shorter than Fz and P3.

### Relationship of responsiveness to observed power changes

As suggested by the significant difference in *k*_e0_ values, the time course of increases in SWA and theta power were also different. The median SWA and beta–gamma increases had a fast onset and rapid decay within about 5 min, whereas the increase in theta power was more prolonged. Five subjects also showed a clear bimodal pattern for theta power (Fig. 2).

Subjects lost responsiveness at 79 (20) s, and regained responsiveness after a wide variation in time (682 [212] s). For the Pz electrode, subjects experienced LOBR on average 33 (32) s before the maximum value of SWA was reached. However, four of them had LOBR around the same time as their maximum SWA, although it is possible that they might have experienced LOBR up to 30 s before, because of the 30 s interval for questioning. The maximum in theta power was significantly later (95 [98] s, *P*=0.04). Maximum beta–gamma power tended to occur close to LOBR (22 [58] s) but was variable with six subjects having maximum beta–gamma up to 46 s before LOBR.

As shown in Table 1, subjects lost responsiveness at higher estimated effect-site concentrations of ketamine than at ROBR (1.64 [0.17] μg.ml^−1^ *vs* 1.06 [0.21] μg.ml^−1^, *P*<0.0001 paired *t*-test). ROBR occurred a long time after the EEG maxima (569 [215] s after the SWA peak, 508 [195] s after the theta peak, and 580 [233] s after the beta–gamma peak). LOBR was associated with increased SWA (13.8 [7.2] dB) than at ROBR (9.3 [2.5] dB, *P*=0.04 paired *t*-test).

**Table 1:** Power (dB) at various time points for the three frequency bands, and concomitant calculated ketamine effect-site concentrations (μg ml^−1^). The power at return of behavioural response (ROBR) is significantly less than at loss of behavioural response (LOBR) for all frequency bands (*P*=0.025 SWA, *P*<0.001 theta, *P*=0.0017 beta–gamma). Power at LOBR and ROBR is significantly less than the maximum power for all wave bands (*P*<0.001). Data for T3 channel are shown. Data shown as mean (sd).

	Gamma	Theta	Slow wave activity	*C*_e_Ketamine
LOBR	–3.09 (2.58)	9.25 (3.52)	12.55 (6.33)	1.64 (0.17)
Maximum power	0.95 (0.99)	25.7 (45.9)	18.1 (0.56)	–
ROBR	–6.56 (2.77)	4.94 (3.79)	7.96 (3.07)	1.06 (0.21)

### PKPD modelling of slow wave activity response

There was inter-individual variation in intensity of response and, on the basis of visual inspection, we removed one channel out of 75 from the SWA model, four from the theta model, and 33 from the beta–gamma model. The fixed-effect model parameters and goodness-of-fit (*R*^2^) for each frequency band and channel are shown in Table 2. The individual time course of the raw and modelled data are shown in Supplementary information. Overall, the model fitted well and comparably for SWA and theta frequencies. The goodness-of-fit was slightly better for the theta frequency band model (*R*^2^, 86 *vs* 80 *vs* 79), but more channels had been withdrawn before the theta model fit than the SWA fit. When the beta–gamma time course was reliable, the model could be fitted well, but about half the beta–gamma records had to be withdrawn from the analysis because they were too noisy to model. Unsurprisingly, the parameters differed significantly between different frequency bands. The effects of channel were only significant for *β*_4_ (i.e. position of sigmoid), which was significantly larger in Pz, *vs* Fz and T3 on *post hoc* analysis.

**Table 2:** Estimated parameters for the pharmacokinetic–pharmacodynamic (PKPD) slow wave activity model from five different channels (Fz, Pz, F3, T3, P3). Values represent mean (standard error of the mean, sem) across individuals. Sigmoid parameters: *β*_1_, baseline activity; *β*_2_, plateau; *β*_3_ and *β*_4_ control the slope and position of the sigmoid. *R*^2^ describes the goodness of fit.

**Slow wave activity**					
Parameter	Fz (*n*=14)	Pz (*n*=15)	F3 (*n*=15)	T3 (*n*=15)	P3 (*n*=15)
*β*_1_	6.99 (0.88)	4.93 (0.81)	2.86 (0.53)	4.46 (0.51)	6.13 (0.46)
*β*_2_	25.40 (4.87)	34.06 (4.78)	20.63 (3.90)	28.83 (3.57)	35.48 (5.54)
*β*_3_	1.72 (0.04)	1.78 (0.04)	1.71 (0.02)	1.79 (0.05)	1.81 (0.05)
*β*_4_	0.05 (0.01)	0.08 (0.02)	0.08 (0.02)	0.14 (0.03)	0.15 (0.04)
*R*^2^	0.79 (0.04)	0.86 (0.02)	0.79 (0.04)	0.77 (0.04)	0.79 (0.02)
**Theta**					
Parameter	Fz (*n*=15)	Pz (*n*=14)	F3 (*n*=15)	T3 (*n*=13)	P3 (*n*=14)
*β*_1_	3.68 (0.92)	2.36 (0.83)	1.85 (0.62)	1.14 (1.64)	1.24 (1.50)
*β*_2_	11.86 (2.56)	14.37 (3.02)	11.14 (2.87)	15.64 (3.70)	18.02 (3.61)
*β*_3_	1.25 (0.12)	1.39 (0.07)	1.55 (0.07)	1.34 (0.16)	1.27 (0.14)
*β*_4_	0.15 (0.06)	0.22 (0.07)	0.16 (0.03)	0.28 (0.07)	0.53 (0.12)
*R*^2^	0.86 (0.03)	0.86 (0.04)	0.87 (0.02)	0.84 (0.02)	0.84 (0.04)
**Beta–gamma**					
Parameter	Fz (*n*=14)	Pz (*n*=5)	F3 (*n*=5)	T3 (*n*=10)	P3 (*n*=8)
*β*_1_	–7.52 (0.77)	–4.66 (0.63)	–3.60 (1.80)	–6.52 (0.83)	–7.49 (0.61)
*β*_2_	11.47 (2.71)	11.78 (1.63)	16.83 (11.70)	8.45 (1.67)	13.8 (4.38)
*β*_3_	1.60 (0.16)	1.87 (0.27)	1.37 (0.08)	1.72 (0.06)	1.69 (0.20)
*β*_4_	0.21 (0.05)	0.30 (0.09)	0.04 (0.02)	0.16 (0.07)	0.23 (0.07)
*R*^2^	0.76 (0.03)	0.67 (0.12)	0.85 (0.04)	0.81 (0.04)	0.74 (0.09)

**Fig 3:**
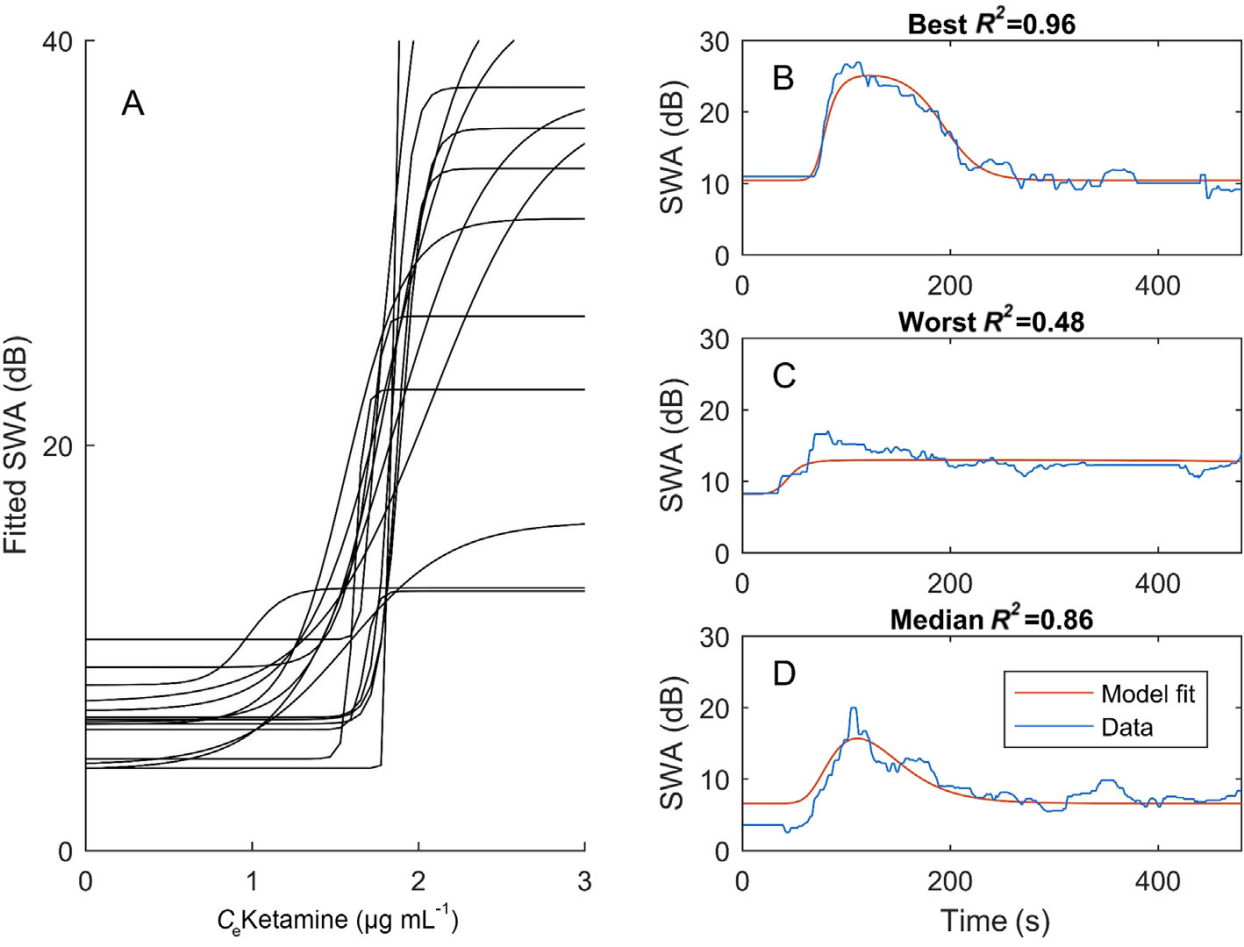
Pharmacokinetic–pharmacodynamic modelling for slow wave activity in the Pz channel. Modelled concentration–effect curves for each subject (a), and examples of the best (b), worst (c), and median (d) modelled slow wave activity (SWA) time course *vs* real SWA time course.

It was rare for the SWA to achieve a plateau at the 1.5 mg kg^−1^ dose of ketamine, so the estimation of the top part of the sigmoid (i.e. *β*_2_ parameter) was least accurate (Fig. 1d). The modelled concentration–response curves, and examples of the best, worst, and median fits are shown in Figure 3. The curves tend to be very steep—saturating over the 1.7–2 μg ml^−1^ concentration range, and reach very high values of SWA for some subjects.

### Slow wave activity morphology

**Fig 4:**
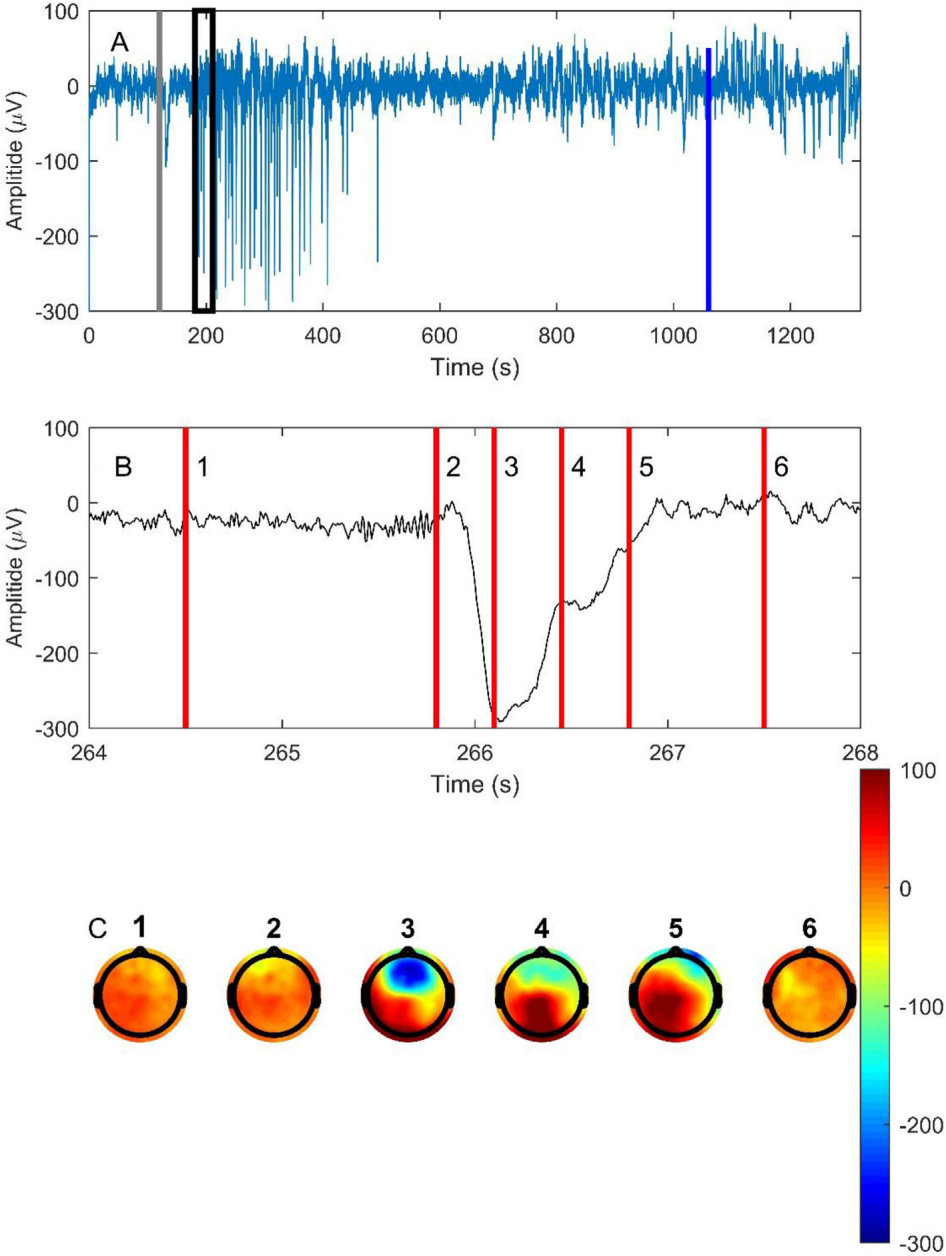
Example of the morphology of ketamine-induced slow waves. (a) Time course of the waves (channel Fz) in relation to ketamine injection (grey vertical line), LOBR (black rectangle) and ROBR (blue line). (b) Close-up view of a single slow wave showing the surrounding theta–gamma oscillations. (c) At the six time points, shown by the vertical red lines, a topological map of the spatial distribution of the instantaneous EEG amplitude is shown. They were chosen as: (1) pre-wave, (2) wave-initiation, (3) maximum-wave, (4) resolving wave, (5) end-wave, and (6) post-wave time points. Colour bar is in units of μV. LOBR, loss of behavioural responsiveness; ROBR, recovery of behavioural responsiveness.

Although our study was not designed as a formal comparison of different SWA morphologies, the slow waves induced by ketamine (Figs. 1d 4) look quite different in shape and size to those typically described during propofol or sevoflurane anaesthesia, or in natural sleep, which tend to be continuous in nature, and smaller in amplitude compared with the episodic ketamine-induced waves described below. Some subjects showed only small, short-lived increases in slow wave power. For those participants who showed an obvious strong response across electrode channels (*n*=9), the slow waves were characterised by between 10 and 45 intense stereotypical hyperpolarisations or depolarisations (absolute amplitude deviation from baseline 140 [58] μV) lasting about 0.3–1 s (median 0.26 s, IQR 0.29 s) and occurring every 3–10 s (median 5.9 s, IQR 6.8 s). These slow-wave episodes occur as interruptions on an underlying ketamine-induced theta–gamma EEG pattern. They start around 40 s after the ketamine bolus and resolve around 300–400 s. Two subjects showed depolarisations that mirrored the hyperpolarisation pattern. These were considered to be a manifestation of a phase reversal/reference contamination phenomenon.^14^ We can see the wave starting in the medial prefrontal region and rapidly enlarging to cover most of the front of the cortex before resolving (Fig. 4b and c). These slow waves are almost always maximal in the midline frontal–prefrontal region (82%). The mirror image (red) 100 μV positive wave seen posteriorly is probably largely an artifact of the average reference montage.

## Discussion

Our results showed that a hypnotic dose of ketamine causes atypical large episodic slow waves that occur around or after LOBR, when the effect-site ketamine concentration was more than ∼1.5 μg ml^−1^. The waves are predominantly medial frontal, and usually consist of large, almost synchronous, hyperpolarisations. These waves were first described by Schwartz and colleagues^1^ in 1974 (see Fig 1, Fig 2 in their paper), but have been relatively ignored since then because they are somewhat obscured by traditional spectral analysis methods that tend to emphasise the obvious ketamine-induced increases in theta and beta–gamma power. The cortical hyperpolarisation pattern seen with these waves has been confirmed using intracellular recordings in cats given ketamine and an alpha-2 adrenergic agonist.^15^

In previous experiments with propofol,^6^ LOBR typically occurred as SWA increased. Mechanistically it is plausible that slow waves cause disruptions in normal cortical function that would interrupt perception. The medial–frontal origin of most of the slow waves is worthy of note. There is some evidence that the medial frontal cortex plays an important role mediating arousal from propofol and volatile agent anaesthesia.^16^, ^17^ Maximal SWA may cause unconsciousness via loss of anterior–posterior functional connectivity, or on the basis of functional MRI evidence of thalamocortical isolation and consequent loss of perception and self-awareness.^6^ It is possible that ketamine reduces consciousness via a similar network-level mechanism. However, the SWA pattern disappeared long before ROBR. This suggests that SWA is capable of disrupting consciousness, but, conversely, the absence of SWA is not sufficient for the return of wakeful connected consciousness. It is likely that the period between loss of the SWA and ROBR was marked by a return of some sort of disconnected consciousness, as manifest by dreaming or hallucinations that are common with ketamine. The effect-site concentration required for LOBR is similar to that found in previous experiments. Schüttler and co-workers^2^ gave five subjects a larger dose of ketamine (250 mg administered as a rapid infusion over a few minutes) to achieve maximal EEG slowing. They achieved peak serum concentrations of ketamine ∼4 μg ml^−1^, and using the median EEG frequency found similar response curves (IC_50_, 2.0 [0.5] μg ml^−1^) for racemic ketamine. Idvall and colleagues^18^ found similar concentrations, and also hysteresis, whereby ROBR occurred at lower ketamine concentrations and smaller SWA than for LOBR.

Flores and co-workers^19^ modelled the time course of high frequency (∼140 Hz) electrocorticogram oscillations in rats after i.p. ketamine. These oscillations showed an initial increase in power that preceded loss of righting reflex, but they did not report on slow waves. Subsequently, ketamine caused a secondary peak in high frequency oscillations that was maximum around the point of regaining righting reflex and outlasted it by about 45 min. They successfully modelled this phenomenon as the mutually antagonistic effects of excitatory *N*-methyl-d-aspartate (NMDA) receptor blocking activity and a second inhibitory (non-NMDA receptor mediated) activity of ketamine. Ketamine has a number of molecular targets, but its actions on hyperpolarisation-activated, cyclic nucleotide-gated subtype 1 (HCN1) currents has been linked to its hypnotic and analgesic effects.^20^ There is increasing evidence that the increase in high frequency power caused by NMDA antagonists is not associated with loss of behavioural response, but is related to psychotomimetic behaviour.^21^, ^22^ Insofar as scalp-recorded gamma activity in humans may be correlated with the very high frequency electrocorticogram oscillations in rats, our results were broadly in agreement with these patterns; and that gamma power should not be used to model drug effects related to LOBR. In our 15 subjects, theta power had an inconsistent relationship with behavioural responsiveness, but probably should be included as part of any subsequent large-scale study of PKPD modelling of ketamine's effects on responsiveness.

The generators of theta waves seen with ketamine are not well understood, and it is not clear why the time course is slower in onset and longer in duration than for SWA and beta–gamma power. We speculate that theta involves slower acting molecular targets and mechanisms downstream from fast ion channels. Increased theta resonance may represent network disequilibrium between anterior and posterior regions with impaired information flow.^4^ This speculative interpretation requires targeted, follow-up investigation (e.g. anatomical source analysis) to advance understanding of such theta oscillations.

There are divergent views on the time course of the onset of the actions of ketamine.^23^ Clinically the time course has been thought comparable with the LOBR seen with propofol and thiopentone.^24^ A detailed study in sheep that compared EEG changes with a mass balance measure of actual brain drug uptake suggested a long *t*_1/2_*k*_e0_ of around 120 s, which is similar to that of propofol.^25^ However, most other studies suggest a more rapid uptake of ketamine to the effect-site. Using the clinical endpoint of LOBR in children, a mean (range) *t*_1/2_*k*_e0_ of 11 (7–20) s was reported.^8^ Our EEG-based estimate agrees with a short *t*_1/2_*k*_e0_ similar to that of methohexitone.^26^

A limitation to this study is that the ketamine plasma levels were not measured directly; accuracy of the model could only be indirectly inferred from dosing and EEG responses. Similarly, it is unclear whether the very steep dose–response curves are a manifestation of a true pharmacodynamic threshold phenomenon (such as a phase-change transition to unconsciousness), or whether they are an exaggerated pharmacokinetic drug diffusion effect caused by the bolus dose. Another limitation is the fact that we had to remove some channels in order to achieve physiologically reasonable model fitting, especially for the beta–gamma analysis. Although not ideal, this does accurately reflect the real-world problems of separating muscle artifact from EEG in a significant proportion of ketamine patients.

## Conclusions

We found that ketamine induced slow wave activity in the electroencephalogram at brain concentrations above ∼1.5 μg ml^−1^, and this was associated with loss of behavioural responsiveness. However, loss of SWA did not correspond to recovery of behavioural responses. As measured by SWA, the time for ketamine diffusion into the brain effect-site (23 s) is much faster than that reported for propofol. The slow waves seen with ketamine are quite different in morphology to those seen with propofol and sevoflurane, and are predominantly medio-frontal in distribution, probably reflecting hyperpolarisations in the medial default mode network (anterior cingulate cortex).

## Authors' contributions

Study design: all authors.

Analysis structure: all authors.

Interpretation: all authors.

Writing of the manuscript: all authors.

Approved of the final version of the manuscript: all authors.

Matlab programming: JS, RP.
