# Supplementary material for: Electroencephalographic slow wave dynamics and loss of behavioural responsiveness induced by ketamine in human volunteers
Source: Br J Anaesth. 2019 Sep 3;123(5):592–600. doi: 10.1016/j.bja.2019.07.021 (PMC6871266; doi:10.1016/j.bja.2019.07.021)

## Slide 1
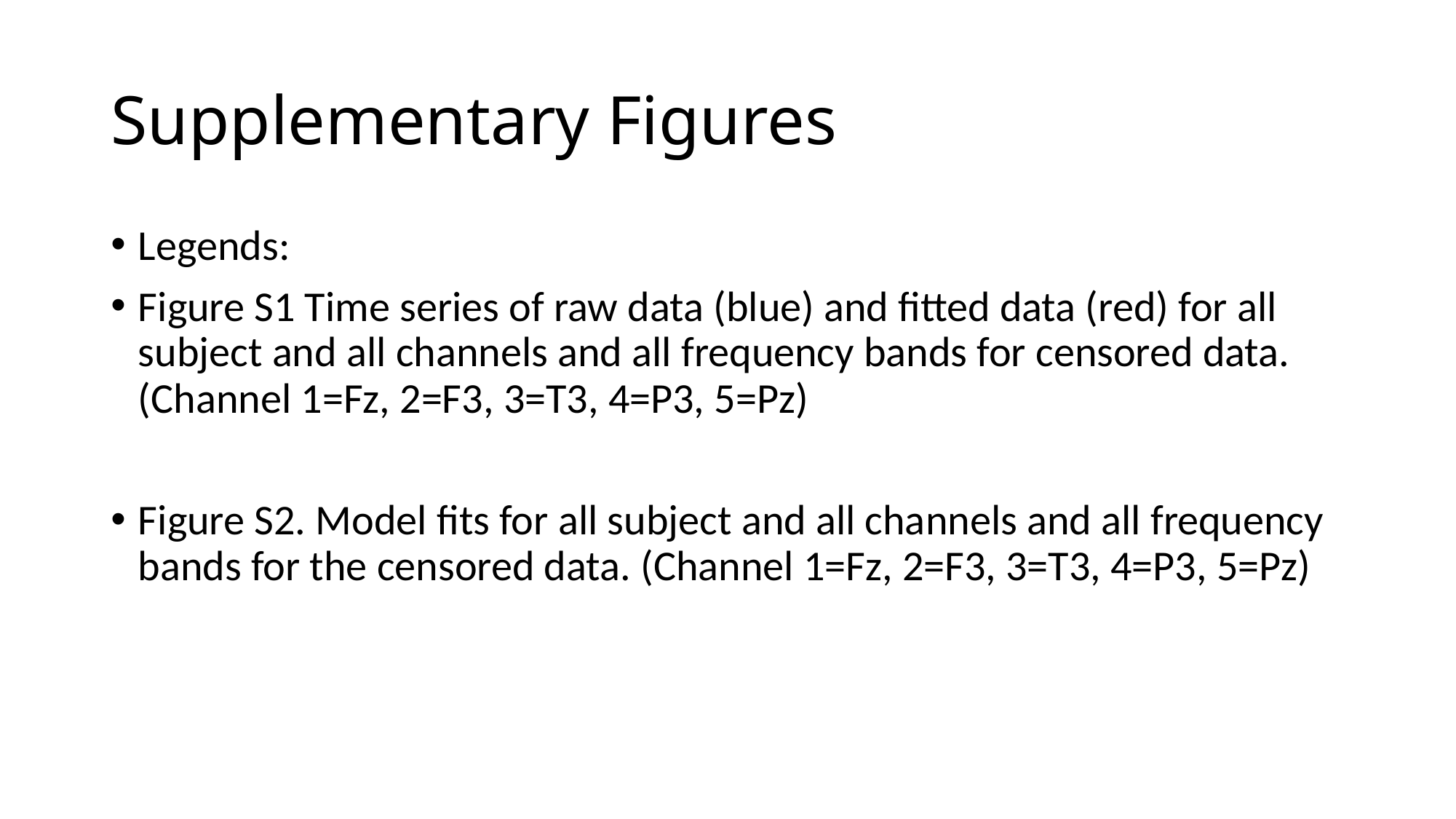

# Supplementary Figures
Legends:
Figure S1 Time series of raw data (blue) and fitted data (red) for all subject and all channels and all frequency bands for censored data. (Channel 1=Fz, 2=F3, 3=T3, 4=P3, 5=Pz)
Figure S2. Model fits for all subject and all channels and all frequency bands for the censored data. (Channel 1=Fz, 2=F3, 3=T3, 4=P3, 5=Pz)

## Slide 2
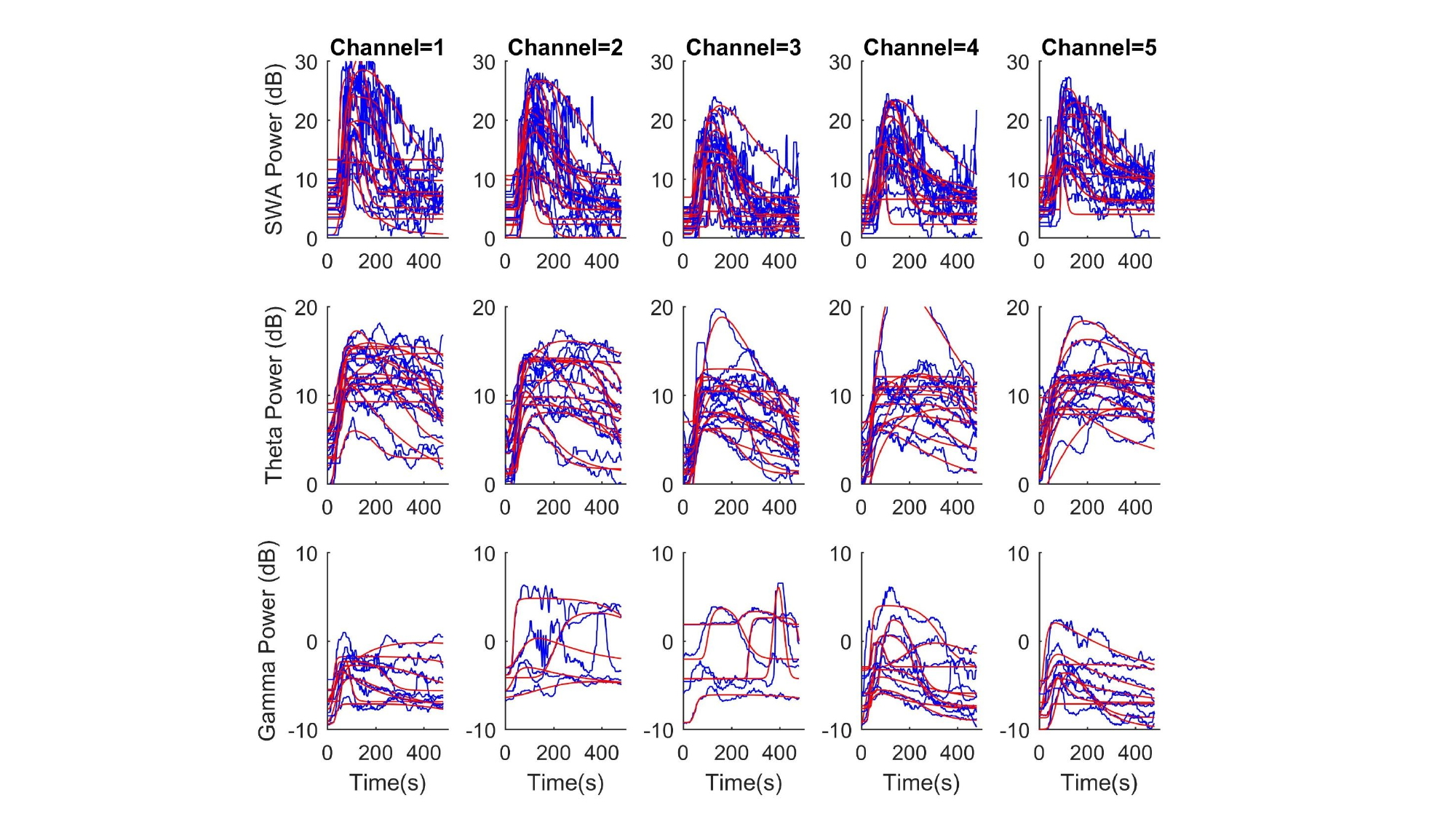

## Slide 3
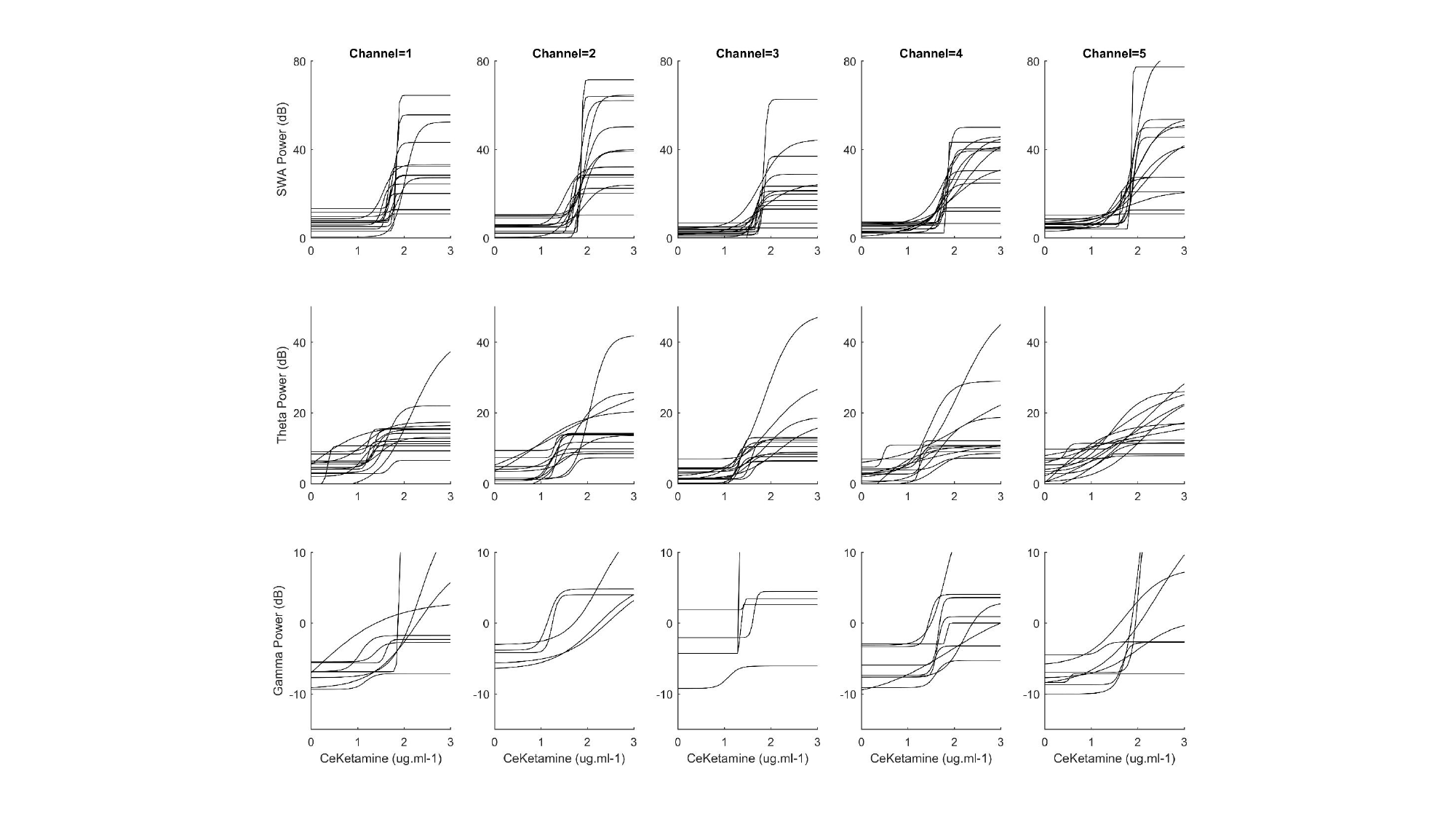

Supplement: Multimedia component 1 [file mmc1.pptx]
